# Basalt Short Fibers Dispersion and Fabric Impregnation with Magnesium Alloy (AZ63): First Results

**DOI:** 10.3390/ma12182960

**Published:** 2019-09-12

**Authors:** Danilo Marini, Marco Valente

**Affiliations:** Department of Chemical Engineering, Materials, Environment, Sapienza University of Rome—INSTM Reference Laboratory for Engineering of Surface Treatments, 00184 Rome, Italy; danilo.marini@uniroma1.it

**Keywords:** basalt, fiber, fabric, magnesium, centrifugal cast, metal matrix composite

## Abstract

Magnesium is one of the lightest structural metals used in different industrial sectors and many works in the literature have studied its reinforcement by filler addition. Basalt fibers are natural fillers that have good mechanical properties, excellent resistance to high temperatures, and lower cost than carbon fibers. Considering this, in recent years, they have been increasingly used in the production of composite materials with polymeric matrices. However, very little information is available in the literature about the use of basalt fibers as reinforcement in metal matrix composite materials. It is well known that the impregnation of fiber reinforcement affects the mechanical behavior of the composite materials. The aim of this study was to investigate the impregnation and the behavior of basalt fibers in a magnesium alloy composite material manufactured by a centrifugal casting technique.

## 1. Introduction

Metal matrix composite (MMC) materials represent a satisfactory solution for obtaining lighter materials with improvement in the typical properties of the metal. Despite this advantage, the production of MMCs still presents some issues that affect the mechanical properties of the final composite in terms of the difficulty of obtaining homogeneous dispersion of reinforcement in the matrix and good impregnation of fillers.

In order to obtain a good interface, it is necessary to evaluate the wettability. Wettability is a property of a solid material regarding the extent to which it can be wetted by a liquid. In this case, the wettability of basalt fibers by molten metal should be evaluated. The wettability between the matrix in the molten state and the reinforcing material depends on several elements, including the intrinsic properties of the material, such as the surface energy of the matrix and reinforcement and the characteristics of the surface of the reinforcement, such as the amount of oxidation and contamination. If necessary, wettability can be improved by increasing the surface energy of the solid; decreasing the surface tension of the molten metal; or decreasing the energy of the particle–matrix interface by coating the particles [1,2], adding additives to the melt, or subjecting it to ultrasonic irradiation [3]. The use of coupling agents improves the wettability of fibers and promotes the formation of bonds to the fiber–matrix interface contributing, for example, to the formation of nonmetallic compounds which, if in thin layers, improve wettability but can damage the surface of the fiber. It has been shown that, depending on the interfacial energies, a metal matrix can incorporate or reject the reinforcement during solidification [4]. Another problem regarding the compatibility of the two phases is the temperature at which the molten metal meets the fibers. If it is too high, in fact, it damages the fibers themselves with consequent repercussions on their mechanical properties. These are the main problems faced in the production of MMCs compared to that of monolithic metallic materials.

Carbon fibers (micro- and nanosized) and glass fibers have been widely studied in the production of polymeric and metallic composite materials thanks to their physical properties (electrical conductivity in the case of carbon nanotubes) and mechanical properties (elastic modulus and tensile strength in the case of microfibers) [5,6].

Basalt fibers are natural fillers that cost less than carbon fibers [7], have very good resistance to high temperatures compared with glass fibers, and have good mechanical properties [8]. Their density (about 2.8 g/cm^3^) is very close to that of glass fibers, although basalt fibers are more resistant [9] and can be used over a wider temperature range, from about −200 to about 800 °C, while type E glass ranges from −60 to 450 °C [7]. Other advantages of basalt fibers are their resistance to alkaline environments, as they can withstand a pH up to 13–14, and their strong resistance to the action of fungi and microorganisms [9]. When in contact with other substances, basalt fibers do not produce chemical reactions that could damage human health or the environment and are incombustible and resistant to explosions. Furthermore, since chemical additives and/or solvents are not added during production, they are 100% natural and sustainable and their recycling is much more efficient than that of glass fibers. When resin composites containing basalt are recycled, these are again obtained in the form of powder because, since their melting point is decidedly high (1400 °C), they are the only product found after incineration [10]. Typical applications of basalt include protective and wear-resistant coatings in tanks, pipes, and pipelines. Basalt composite pipes, in fact, can be used for the transport of petroleum products and aggressive liquids and the supply of hot and cold water [11]. Furthermore, they are ideal for fire protection, as fireproof materials in nuclear power plants, and electrical insulation applications, for example, in printed circuits [11].

Despite this, very little information is available in the literature about the production of basalt-fiber-reinforced MMCs.

In this work, we focused on manufacturing composite materials with a magnesium alloy matrix and basalt fiber reinforcements by induction melting [12] and centrifugal casting.

In particular, the aim of this study was to evaluate the impregnation of basalt fibers by a magnesium alloy and to compare the behavior of basalt microfibers (BMFs) and basalt woven fabrics (BWFs) in the magnesium alloy matrix produced by the manufacturing process previously mentioned.

## 2. Materials and Methods

A typical magnesium foundry alloy was selected as the matrix for composite manufacturing: AZ63 alloy shavings, containing 6–7 wt % of aluminum and 3–4 wt % of zinc. Shavings were provided by COMETOX (Italy) and used without further purifications (Table 1).

Milled recycle BMFs (300 µm in medium length and 18 µm in medium diameter) produced by BASALTEX were selected as the short fillers and their characteristics are reported in Table 2.

**Figure 1:**
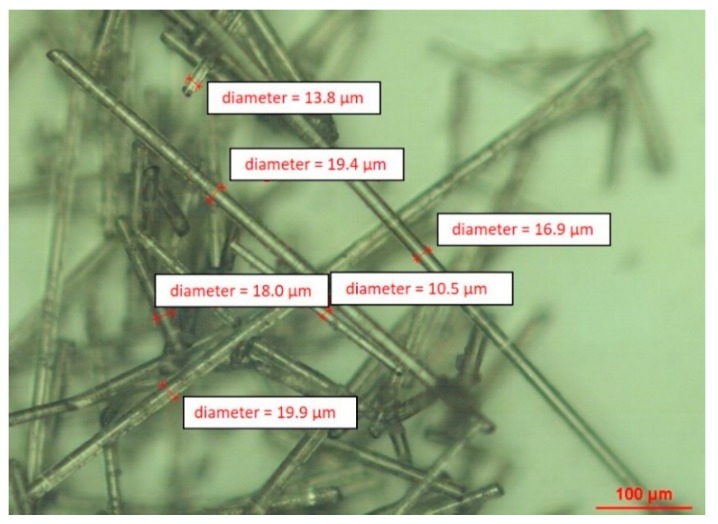
Milled recycled BMFs.

This type of basalt fiber was used as received. The actual fiber diameters were measured by optical microscopy (Figure 1).

A BWF produced by BASALTEX (Gullegem, Belgium), with a specific surface weight of 220 g/m^2^ and a silane sizing agent designed to be compatible with epoxy and thermoplastic resin matrix, was used as the long filler. This type of basalt fiber has a nominal diameter of 13 µm, and the actual fiber diameters were checked by optical microscopy.

Thermal treatment results in degradation of the fiber sizing [8], and in this work, manufactured composite samples using BWFs were used after thermal treatment in air at 400 °C for 4 h to avoid sizing problems in terms of adhesion and bubble production at the melting temperature of the magnesium alloy.

**Figure 2:**
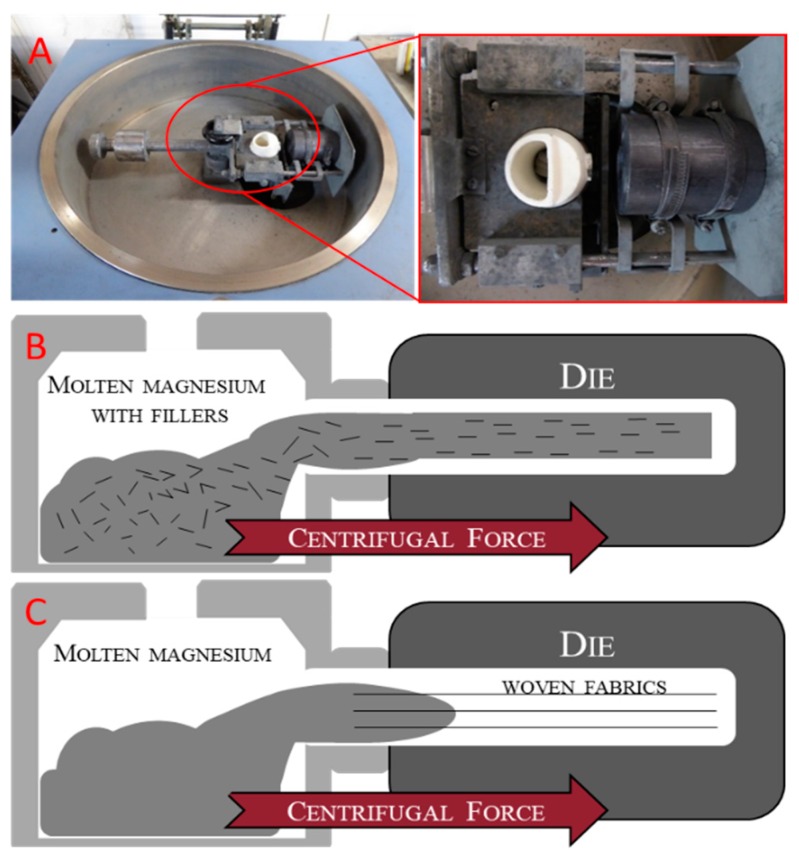
(**A**) Induction melting and centrifugal casting furnace. (**B**) Schematization of method used with short basalt fibers. (**C**) Schematization of method used with basalt woven fabric.

Magnesium composites with short fillers were prepared by mixing the metal shavings with different weight percentages of BMFs in a mortar using acetone to facilitate the dispersion of fillers in the metallic shavings. This mixture was charged in a melting pot and prepared to be melted and cast (Figure 2).

Magnesium composites with long fillers were manufactured by charging only shavings in the melting pot, while the BWFs were positioned and fixed directly in the mold cavity section in a longitudinal position with respect to the casting axis (Figure 1). This configuration allowed for obtaining the impregnation of basalt fabrics by the melted magnesium alloy.

In both cases, melting pots (mod. C20) and a graphite mold with a parallelepiped cavity (10 × 5 × 50 mm) produced by F.lli Fossati (F.lli Fossati S.r.l., Milano, Italy) were used.

Both kinds of samples were produced by a Neutor Digital F.lli Manfredi (F.lli Manfredi Saed S.r.l., Torino, Italy) induction furnace that allowed melting under a controlled atmosphere (Ar) and casting with centrifugal force (Figure 2).

Table 3 lists the melting and casting parameters.

To evaluate the influence of the reinforcement ratio, in the case of short fibers, two different percentages of fillers were adopted (5 and 10 wt %), while for composites with long fillers, one or two layers of BWF (≈5 and 10 wt %) were inserted in the composites.

**Figure 3:**
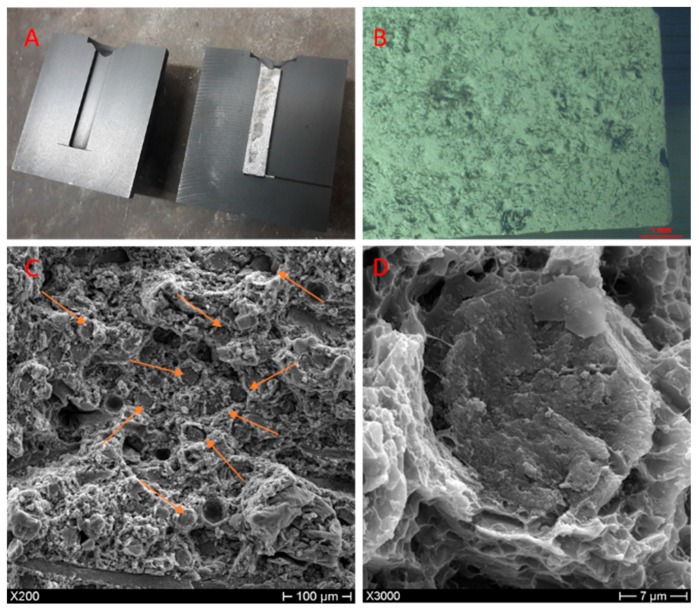
(**A**) Sample after casting with short fillers. (**B**) Cutting surface after acid treatment. (**C**) Fracture surface of sample with short fillers. (**D**) Enlargement of one single short basalt fiber.

**Figure 4:**
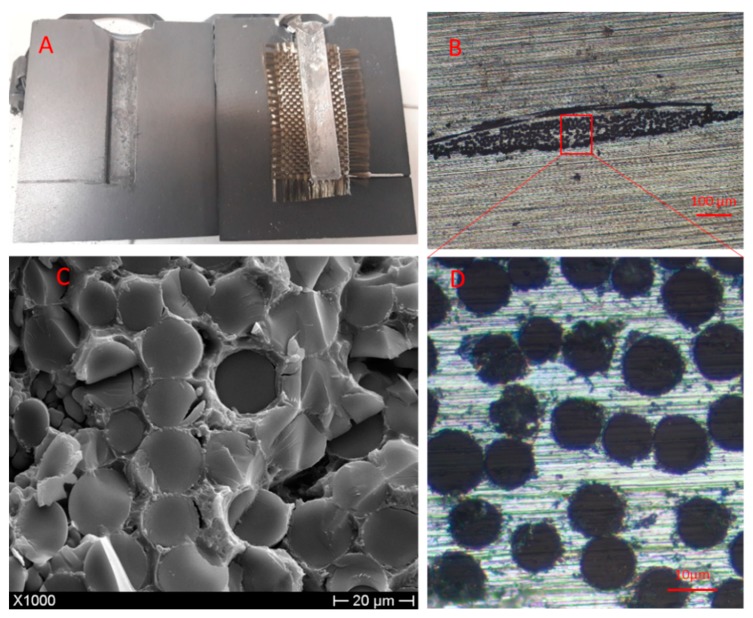
(**A**) Sample after casting with long fillers. (**B**) Cutting surface of sample with long fillers. (**C**) Fracture surface of sample with long fillers. (**D**) Enlargement of cutting surface in (**B**).

Figure 3 and Figure 4 show the raw samples obtained when opening the die after mixing and casting of the composites reinforced with BMFs and impregnation of the BWF, respectively.

Two different castings for each kind of sample were carried out. Raw samples obtained were cut longitudinal to the casting axis and perpendicular to the woven fabric in order to obtain two samples for each casting. The rods obtained (4 × 3 × 50 and 4 × 2 × 50 mm) for the short and long fibers, respectively, were prepared for four bending tests [13,14] at room temperature with a Zwick Roell Z2.5 testing machine (Zwick Roell, Ulm, Germania). Before mechanical tests, the porosity of the samples was evaluated by density measurement using a ME54 Mettler-Toledo (Mettler-Toledo, Milano, Italy) analytical balance, equipped with a tool for solid density measurements by the Archimedes principle.

The residual porosity of the composites was calculated by comparing the theoretical and measured density of the samples.

The comparison between the theoretical and measured density of the manufactured composites was calculated with Equation (1):

(1) $$\mathrm{Porosity}\%\;=\;\frac{\rho \mathrm{th}\;-\;\rho \mathrm{me}}{\rho \mathrm{th}}\;\times \;100$$

where ρth is the theoretical density calculated with Equation (2), and ρme is the measured density using the Archimedes method mentioned above.

The theoretical density was calculated with the rule of mixtures (Equation (2)):

(2) ρth = ρfVf + ρmVm

where ρth is the composite’s theoretical density, ρf is the fiber density, ρm is the matrix density, Vf is the volumetric ratio between the fibers and the composite, and Vm is the volumetric ratio between the matrix and the composite.

The cutting and fracture surfaces were analyzed by optical microscopy and SEM (Figure 3 and Figure 4), performed using a NIKON–L150 (Nikon, Tokyo, Japan) and a PHILIPS XL-40 (FEI B.V, Eindhoven, The Netherlands), respectively, in order to evaluate the dispersion and impregnation of the fillers.

## 3. Results

Optical microscopy analysis revealed the presence and good dispersion of the BMFs in the AZ63 specimens. Figure 3 shows, as an example, an image of the sample at 5 wt % after acid attack with concentrated HCl, which was done to make the fibers visible. SEM analysis of the fracture surfaces confirmed the optical analysis results. Moreover, Figure 3 shows that the BMFs were well wetted by the metal matrix and partially aligned in the direction of the casting axis; however, more defects and greater porosity were visible on the fracture surface. The presence of residual porosity was also confirmed by the results obtained from the comparison between the theoretical and measured density. The diagram in Figure 6 shows the comparison between the theoretical and measured density of the manufactured composites, and the composites reinforced with 5 or 10 wt % of both kinds of filler showed, respectively, the residual porosity (reported in Table 2).

These values were comparable to the residual porosity measured in the samples without basalt reinforcement and, therefore, intrinsic to the chosen manufacturing process.

Comparing the results of the SEM analysis reported in Figure 3 and Figure 4, it is possible to see the different morphologies of the fibers in the fractural surface. The composites reinforced with BMFs (Figure 3) showed greater fiber degradation than the composites reinforced with BWFs. This effect can be attributed to the longer residence time of the short fibers at high temperatures [15], which were mixed with the metal directly in the melting crucible, compared with the fabrics, which were impregnated by the liquid metal in a few seconds.

**Figure 5:**
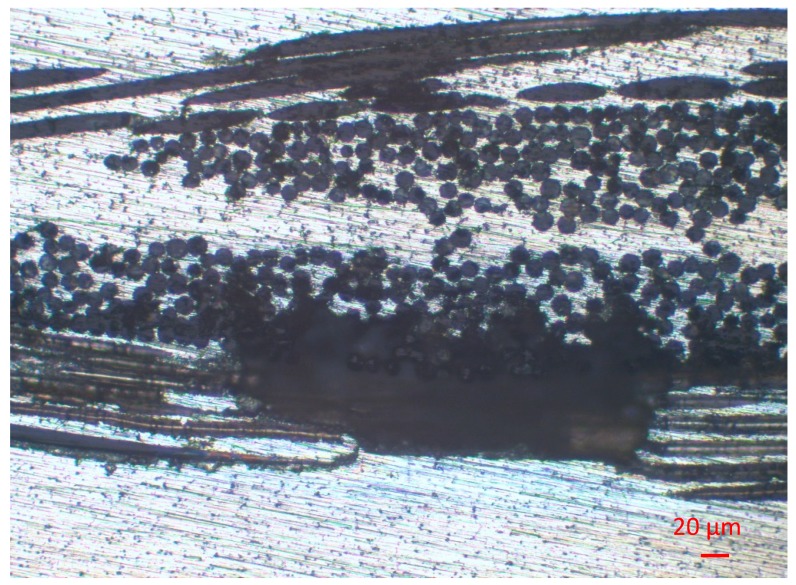
Nonhomogeneous impregnation of basalt woven fabric (BWF) by molten AZ63.

Optical and SEM analyses of the BWF samples showed good impregnation of fibers by the metal matrix, but this was not homogenous throughout the sample’s length. Figure 5 shows one of the many parts of the BWF not completely impregnated by the metal of the matrix. Figure 4, however, demonstrates that for the cutting surface of the single-layer composite, the metal had completely penetrated between the threads of the fabric.

Figure 4 shows a fracture surface, in which it is possible to observe the clear fracture of the basalt fibers with the complete absence of pullout phenomena, indicating an excellent matrix–fiber interface with a complete transfer of the applied load from the matrix to the reinforcements in these parts of the composite. In this picture, different longitudinal fractures on the fibers are also visible, which were probably due to the thermal shock caused by the casting of molten metal.

**Figure 6:**
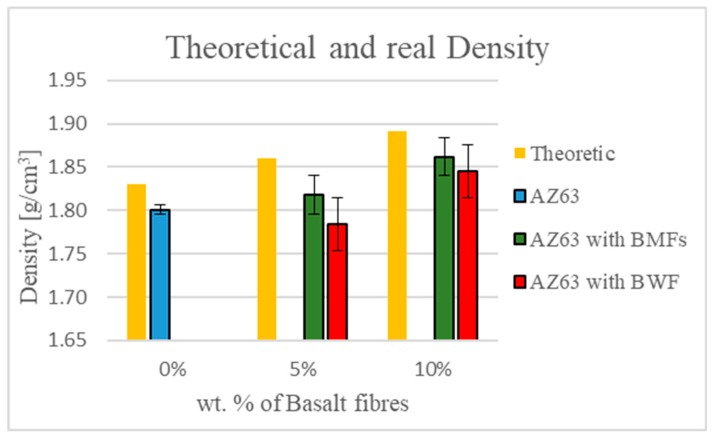
Comparison of theoretical and measured density of composites obtained.

The results reported in Figure 6 and Table 4 also confirm the optical microscopy and SEM analyses. The residual porosity had already been detected in the non-reinforced AZ63 and the AZ63-BMF samples, but a higher residual porosity was present in the composites reinforced with BWF, probably due to the nonhomogeneous impregnation of the fabric. The residual porosity produced effects on the final mechanical properties of the composites, the results of which are reported below.

**Figure 7:**
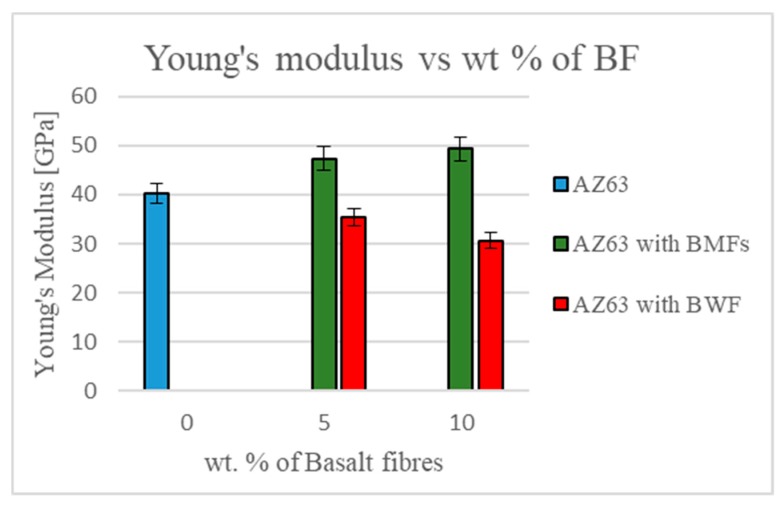
Young’s modulus for the two kinds of samples.

Mechanical characterization was performed on the two types of manufactured composites, and the Young’s modulus, in the case of the BMFs, increased along with the filler concentration (Figure 7). The flexural strength increased for 5 wt % BMFs but decreased along with the filler concentration because of the number of defects in the matrix, probably due to the effect of the addition of BMFs on the rheological property of the mix, which were pushed into the die only by centrifugal force.

**Figure 8:**
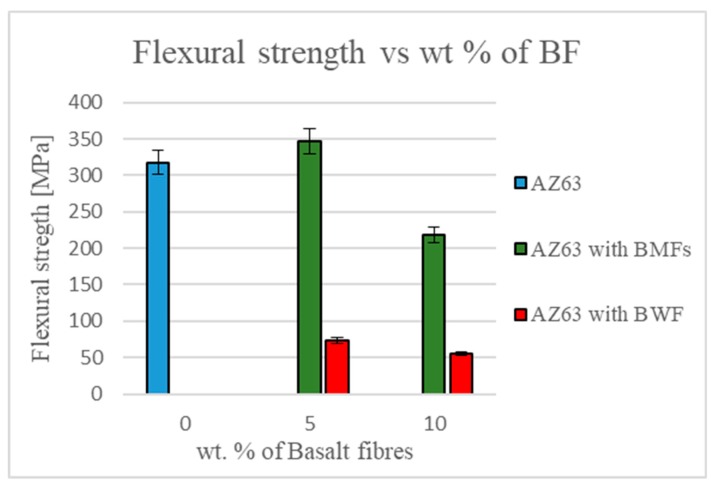
Flexural strength trend for the two kinds of samples.

Mechanical tests on the composites with BWFs showed a decrease of Young’s modulus and a drastically decreasing of flexural strength, probably due to the embrittlement by thermal shock of the fibers and to the nonhomogeneous impregnation of the fabric (Figure 8).

## 4. Conclusions

In this work, the interaction between a typical magnesium alloy (AZ63) and basalt fibers in two different configurations (short fibers and woven fabric) was studied in order to evaluate the possibility of producing MMC materials with a particular manufacturing process based on induction melting and centrifugal casting.

The results of the SEM observation along with the density measurement performed on the composites reinforced with BWFs confirmed the presence of higher residual porosity in this type of composite than in the AZ63 samples and the composites obtained with BMF reinforcement.

Despite the difficulty and the residual porosity measured in the sample, the results showed good interfacial interaction between the matrix and the fillers, in particular in the case of composites with the addition of 5 wt % of BMFs. In fact, at the expense of a weight increase of only 0.92%, we found an increase of Young’s modulus of around 20% and an increase of flexural strength of 10%.

From the results obtained, basalt fibers can be considered as a potential reinforcement for magnesium alloys thanks to the good impregnation and strong interfacial interaction between them.

The centrifugal casting manufacturing process needs some optimization in terms of operative parameters, principally to eliminate the residual porosity, which was detected in the samples produced, and to obtain optimal impregnation of the BWF.

Preheating the mold with the fabric inside and varying the rotation speed of the centrifugal casting system are two possible strategies to optimize the manufacturing process to avoid the effect of thermal shock and to ensure good fluidity of the molten metal during the casting process, respectively.

## Figures and Tables

**Table 1:** AZ63 characteristics.

Parameters	Value
Melting temperature (°C)	610
Specific gravity (g/cm^3^)	1.83
Tensile strength (MPa)	300
Young’s modulus (GPa)	40

**Table 2:** Basalt microfiber (BMF) characteristics.

Parameters	Value
Melting temperature (°C)	1500
Specific gravity (g/cm^3^)	2.7
Tensile strength (MPa)	2800
Young’s modulus (GPa)	88

**Table 3:** Melting and casting parameters.

Parameters	Value
Melting temperature (°C)	660
Melting time (s)	60
Absolute pressure (mbar)	800
Inert gas	Argon
Rotation speed (rpm)	350

**Table 4:** Porosity percentages in the manufactured samples.

Filler Ratio	AZ63	AZ63-BMFs	AZ63-BWF
0 wt %	1.58%	-	-
5 wt %	-	2.28%	4.06%
10 wt %	-	1.53%	2.40%
